# LONG-TERM EFFECT OF A MULTICOMPONENT INTERVENTION ON PHYSICAL PERFORMANCE AND FRAILTY IN OLDER ADULTS

**DOI:** 10.1093/geroni/igz038.3351

**Published:** 2019-11-08

**Authors:** Gahee Oh, Il-Young Jang, Heayon Lee, Hee-Won Jung, Eunju Lee, Dae Hyun Kim

**Affiliations:** 1 Harvard T.H. Chan School of Public Health, Boston, Massachusetts, United States; 2 Division of Geriatrics, Department of Internal Medicine, Asan Medical Center, University of Ulsan College of Medicine, Seoul, Korea, Republic of; 3 Seoul National University Hospital, Seoul, Korea, Republic of; 4 Division of Gerontology, Beth Israel Deaconess Medical Center, Harvard Medical School, Boston, Massachusetts, United States

## Abstract

Multicomponent interventions improve physical function and frailty in older adults, but their long-term benefit remains uncertain. We report the 30-month outcomes of a 24-week multicomponent intervention versus usual care in community-dwelling older adults. This prospective non-randomized study was conducted in 383 older Koreans (mean age, 76.8 years; female 72.3%) living alone or receiving medical aid in rural communities. Of these, 187 received a 24-week multicomponent intervention that consisted of group exercise, nutritional supplements, depression management, deprescribing, and home hazard reduction. The remaining 196 individuals received usual care. After 1:1 propensity score matching, we compared the short physical performance battery (SPPB) score (0-12 points), frailty phenotype scale (0-5 points), and deficit-accumulation frailty index (0-1) at 6, 18, and 30 months. Restricted mean survival time was estimated for death and institutionalization-free survival time at 30 months. The intervention group had higher SPPB scores than the comparison group at 6 months (difference 3.2; 95% CI, 2.5-3.8), 18 months (1.2; 95% CI, 0.5-1.9), and 30 months (1.1; 95% CI, 0.5-1.8). They had lower frailty phenotype scale (-0.6; 95% CI -0.9 to -0.3) and frailty index (-0.04; -0.07 to -0.02) only at 6 months, but similar scores at 18 and 30 months. Death and institutionalization-free survival time over 30 months was 28.5 months (95% CI, 27.7-29.3) in the intervention group versus 24.2 months (95% CI, 22.3-26.1) in the comparison group (difference, 5.2 months; 95% CI, 3.0-7.3). The 24-week multicomponent intervention showed sustained improvement in physical function for 30 months, but only temporary reduction in frailty.

